# Ethical framework of assistive devices: review and reflection

**DOI:** 10.1186/s40638-017-0074-2

**Published:** 2017-11-15

**Authors:** Nazanin Mansouri, Khaled Goher, Seyed Ebrahim Hosseini

**Affiliations:** 1 Department of Land Management and Systems, Lincoln University, Lincoln, Canterbury, 7647 New Zealand; 2 School of Life & Health Sciences, Aston University, Birmingham, B4 7ET UK; 3 Faculty of Environment, Society and Design, Lincoln University, Lincoln, Canterbury, 7647 New Zealand

**Keywords:** Robot ethics, Assistive medical robots, Robot ethics framework, Older adults, Assistive walking device framework, Ethics concerns, Ethics issues

## Abstract

The population of ageing is growing significantly over the world, and there is an emerging demand for better healthcare services and more care centres. Innovations of Information and Communication Technology has resulted in development of various types of assistive robots to fulfil elderly’s needs and independency, whilst carrying out daily routine tasks. This makes it vital to have a clear understanding of elderly’s needs and expectations from assistive robots. This paper addresses current ethical issues to understand elderly’s prime needs. Also, we consider other general ethics with the purpose of applying these theories to form a proper ethics framework. In the ethics framework, the ethical concerns of senior citizens will be prioritized to satisfy elderly’s needs and also to diminish related expenses to healthcare services.

## Introduction

Ethnographic reports present that ageing population is growing significantly all over the world [1, 2]. This increase gives rise to particular needs of elderly people [3–5]. Moreover, population increase leads to substantial issues such as shortage in medical centres, healthcare services, and medical professionals [6] and burdens of enormous amount of healthcare expenses [7]. Recently, there have been noticeable technological innovations in Information and Communication Technology (ICT). These developments have resulted in creation of various types of assistive medical robots such as RIBA, paro-robot, telerobot, and remote presence robot [7], assistive devices, home automation systems, and canes [8–11]. Assistive devices and robots are developed with the purpose of fulfilling elderly’s needs and expectations, compensating their disabilities, boosting their life quality, providing assistance to carry out task(s), whilst maintaining their autonomy [7, 12]. Research studies revealing the primary needs of older adults are listed in Table 1 [13, 14]. Medical assistive robots including walking devices can be adopted by elderly if they prove to be useful, reliable, efficient, effective, and also easy to utilize [15, 16].

**Table 1:** Primary needs of elderly

Primary needs of elderly
To stay in their own places, whilst keeping their independency and quality of their lives safely
To grow medical professionals and doctors attention towards elderly’s well-being
To have control on their own life during course of emergency requiring assistance
To be motivated to take part in community life with the purpose of alleviating negative feelings, namely social isolation

### Paper organization

This paper is organized as follows: “Theories of Ethics” section introduces a summary of literature of general ethics, human rights and values; “Ethical Issues of Assistive Medical Robots” section addresses existing ethical issues related to assistive medical robots; “Discussion and Conclusion”, summarizes the important role of ethical framework on both assistive medical robots and walking devices

## Theories of ethics

Under this section, general ethics theories as well as human rights and values are described.

### General theories of ethics and bases of ethics concerns

In area of ethics, there are general theories which can be practiced in different fields such as assistive medical robot including walking devices ethics. Robots, specifically assistive medical robots, simulate human behaviours, performance, and actions; therefore, ethics general theories can be applied in design and use of medical robots like walking devices. The prime example is the robot’s program where the program’s codes are written based on ethics general theories, whilst taking ethics concerns into consideration. This section of the paper describes three (3) relevant general theories of ethics and also bases of ethics concerns.

#### Deontology

The word “deontology” is taken from two (2) Greek words which are duty and study. Deontology ethics, which is established by Immanuel Kent, is known as non-consequentialist or duty based [17]. According to this theory, individuals are enforced morally to perform or take actions in accordance with series of principles as well as rules without considering the outputs of taken actions [18]. This theory mainly considers rightness and wrongness of an action itself rather than focusing on its consequences and outputs [19]. In accordance with Sullivan [20], this is the first ethics theory prioritizing decision-making to a person. Moreover, [21] stated that in a moral action of an individual, feelings and incentive refuse to play a significant role. Therefore, incentive for taking an action is based on obligation before the action takes place [17]. In other words, in accordance with this theory, in spite of destructive consequences, individuals are required to take right actions which are based on rules [22].

The prime example of applying duty-based theory in assistive medical robots is giving medicine to an older adult. If a senior citizen requests a painkiller from his/her assistive medical robot, in spite of being allergic to the painkiller, the medical agent is required to follow rules and to provide the medication to older adult. It is evident that medical robot action triggers older adult’s health condition. In contrast, in other general theories of ethics such as consequentialism, the action of medical robot endangering older adult’s well-being declines to be accepted; therefore, assistive agent is required to provide another solution to relive older adult’s discomfort.

#### Virtue

This ethics virtue is recognized as character-based ethics which is far towards individual based rather than action based. Virtue ethics is recognized a character-based ethics highlighting an individual’s right action in all the same circumstances [23]. This theory emphasizes on virtue and moral character of a person carrying out an action rather than considering action’s consequences or ethical rule [24]. The concern of this ethics theory is not only focusing on rightness or wrongness of an individual’s action but also offers a number of behaviours.

Virtue theory is beneficial if an individual incline to assess another individual character rather than goodness or badness of a particular action. In this theory, individuals are required to have series of characteristics for virtuousness [25].

Character-based theory sporadically tends to deontology ethics theory, whilst it is contrary to consequentialism ethics theory. The prime example is helping needy: based on consequentialism theory, helping needy improves well-being. On the other hand, deontology theory says that helping needy is in accordance with moral rule, whilst virtue theory argues that this kind of assistance is a character of generosity.

#### Consequentialism

This ethics theory is known as result-based theory which highlights two (2) primary principles. The first one states that rightness or wrongness of an action is based on its result and potential consequences. The second concept indicates that when the result of an action has greater consequences, that action is considered as a more right action [26]. In accordance with consequentialism, an action is favourable if its consequences refuse to produce harmful consequences.

Hedonism and utilitarianism are two forms of consequentialism ethics theory. Hedonism indicates that it is necessary for individuals to ameliorate human, whilst utilitarianism states that it is essential for individuals to enhance human health. In addition, another form of consequentialism states that individuals are required to improve their preferences satisfaction and happiness.

It is stated by Cummiskey [27] that in result-based theory a murder is considered right if its consequences produce good result. In other words, if a murderer inclines to kill a group of innocent individuals, it is accepted based on consequentialism to kill the murderer to save the victim’s lives. In contrast, based on both deontology and virtue theories, in spite of victims death, killing the murderer is wrong [28].

#### Human rights and values

Human rights related to senior citizens consist of the right to a standard of living which is sufficient for health and welfare, freedom from discrimination, inhuman and torture or humiliating treatment, and private and family life.

A focus on human rights provides support to highlight that physical and psychological well-being of older adults is as significant as the well-being of other member of society. Therefore, it is substantial to make sure those assistive medical robots embedded into older adults lives aim at benefiting elderly, and not embedded to diminish care burden on the other people [29]. In addition, it is essential to consider twelve human values which are introduced to technological developments [30].

## Ethical issues of assistive medical robots

The debates about the ethical actions of robots date back to 70 years ago [31]. From 1950, when Asimov presented his three laws, [33] there has been arguments about the potency of those particular rules to render robots capable of making ethical decisions independent of human interference. The key argument of Asimov’s laws considered the self-directedness of robots. Being autonomous, robots were assumed to have the physical and intellectual capacity to make moral decisions, using the knowledge and rationality which they were equipped with [34]. Asimov’s three laws discussed these notions: (1) A robot may not be a source of damage for a human being or, its inactivity expose a human being to harm. (2) A robot must follow human beings’ orders except the ones which would confront the first law. (3) A robot should guard itself as long as such defence does not contrast with the other two laws.

Both researchers and science fiction writers have expressed their concerns about a number of ethical issues that daily use of robots has made them possible. However, the robots that we use daily are limited to vacuum cleaners, grass cutters, and robot toys. These are not same to the advanced science fiction robots that are the subject of the recent robotics ethics [32]. Consequently, the ethical concerns related to robots should not be based on empirical data and studies done by users. Instead, taking Asimov’s laws as an opening point for ethical debates [35], they need to discuss ethics according to their potential, future application [36].

Robots-exclusive Concerns. Ryan Calo is a law professor who wrote the “Robots and Privacy” chapter in Robot Ethic. He points out that the debated on robots are currently paying attention to ubiquity, and, conceivably, this is not that good [37]. Calo detects three privacy dangers which robots can create: “surveillance”, “access to living and working spaces”, and “social impact”. The anxiety about such an access is exacerbated by the research done by Denning et al. [38]. In this research, the authors explore vulnerable security measures in several toy robots [37].

Certainly, in areas such as robotics, producers need to be very innovative. Current world is witnessing a technological explosion with new possibilities. One argument is that ignoring speculation about future robots and their use can create ethical dilemmas. However, our argument is that it is necessary to adapt a perspective that is in agreement with the experiences resulted from empirical use of robots. This will help to complement the current debate on robot ethics.

A list of ethical issues related to the use of assistive medical robot from older adults’ perspective are explained in details in this section and the following section. There are a noticeable number of ethical issues which are stated by senior citizens about the use of assistive medical robots. Amongst the stated issues, there are primary issues which are of significant concern not only to the older adults but also to elderly’s family and caretakers, robot designers and developers.

Moreover, trust is a vital element for the formation and preservation of humans’ dynamic relationship with assistive robots [39–41].

The lack of trust is the main reason that seniors do not wish, do not need, or do not consider robots.

Lack of trust results from some factors [42, 40]:

- Privacy: how can youth and the elderly leave their privacy in the hands of a robot?
- Safety: If a robot is set to undertake physical responsibilities, the physical interaction of human–robot leads to serious challenges. Besides, upgraded methods are necessary to eliminate the failures raised by safety problems and confirm the absence of any unreliable behaviour.
- Robustness: despite the circumstances, how the elderly can be convinced about the suitability of the behaviour of a robot?
- Security: Affirming that the robot is not harmful for the elderly.
- Data protection: how can the elderly be convinced of the safety of the significant data?

The ethical issues of assistive medical agents are listed in sections in below.

### Privacy of older adults

Privacy of senior citizens is of paramount importance that it is well in line with other ethical issues such as data protection and security and safety. This ethical issue is of great concern to scholars [43–52]. This issue has substantial effect on older adults to lose their appeal to adopt smart home technology. The main process of smart home technology includes collection, transmit, distribution, and exchange of elderly’s private information. This main process has impact on elderly to refuse smart home technology [43, 46, 53, 54]. Take home healthcare robots as an example; this kind of robot enable medical specialists to keep a wary eye on their patients’ well-being in remote places by means of various tools such as camera, ultrasound, and speaker [7].

The process of Ambient Intelligent Technology (AIT) consists of various procedures such as collecting, distributing, and storing full confidential data of user [55]. The key functions of this technology are to keep eyes on robot’s user and to combine data from different resources by sensors to obtain the details of circumstance [45]. In the process of data collection, profound, medical, and confidential data of robot user are gathered. In addition, other parties might have access and control to the gathered data; therefore, user’s privacy might be abused [55, 56].

In addition, home automation system is one of the main ICT devices employed for fall prevention purpose. Home automation system is type of device which is wearable attached to the body of user by means of transparent film and neoprene belt. The primary function of this device is to detect fall incidents through video monitoring [9, 10]. It is asserted from various studies that noticeable numbers of senior citizens are of critical concern about their privacy. Consequently, it is far favourable for them if the wearable device captures unclear photographs when they are at personal places such as bedroom. In contrast, it is accepted for elderly if the device takes clear images when they are at other rooms such as living room [57, 10]. It is claim that privacy concern slackens older adults’ interest towards this kind of devices especially visual surveillance or cameras [58].

Two-way visual contact is a way of communication and connection through webcams and television monitors, though it is not widely used despite its rather cheap price. This allows family members or employed carers to “look in” on older persons and their homes with no need to commute [59]. If older people feel at ease in working with computers, virtual visiting and communication is reasonable and easily established. It is not more difficult than installing and making a Skype account. Even there are virtual visiting systems which are more user-friendly than Skype and operate by connecting to local broadband networks.

### Data protection

The ethical issue of data protection is well connected to privacy issue. In the process of home healthcare services, there should be a connection between both medical centre personnel and the place of robot user to provide not only safety services but also social care and daily basis services [49]. In multi-user cases, the intelligent system is in charge of distinguishing different data, namely robot user’s private data, caretaker’s data, as well as other relevant information to monitor well-being of user [60]. Consequently, it is essential to subject the collected data to act of data protection [61].

The primary function of assistive walking devices including fall detection devices such as home automation systems is to capture image or record video of older adults. The captured images or recorded videos might be inappropriate or unwanted; therefore, these images or video are unfavourable to elderly. Moreover, it is of importance concern to older adults if their personal data, namely images or videos, are accessed and viewed by third parties.

Some assistive robots are used to help in remote sensing and monitor the elderly in variety of locations. These robots are as assistant for those specialists who want to check their patients remotely, mainly in critical situations. They do this by making use of speakers, light, cameras, remote controls, ultrasound, and electronic medical recording accessories [62].

### Security and safety

Safety and security ethical issue is well related to privacy concern [63]. It is strongly recommended that there should be a balance amongst the needs of elderly for safety, whilst preserving elderly’s privacy and autonomous [29, 56, 64]. In addition, it is claimed that older adults, their families and caretakers have contrary point of view about privacy, safety and security concerns [56]. A conducted study reveals that family and caretakers of senior citizens are more concern about safety and security rather than privacy and independency concerns [56]. Moreover, although some scholars subscribe to the belief that there should be a balance between privacy and safety concern [29, 64], other scholars believe that safety and security of elderly is of dramatic importance [43, 65–67].

Regarding security and safety of walking devices, over recent decades, one of the substantial and pricy public health issues is fall incidents and injuries happen to older adults [68–70]. It is found that one older adult out of three with the age of sixty-five or above falls yearly resulting in serious injuries which require treatment in medical centres [71–73, 69]. Although there have been significant developments in fall prevention devices, fall incidents take place with severe consequences such as morbidity and mortality. Injuries resulted from fall incident are ranked number five in terms of causing mortality in ageing group with the age of sixty-five and above [71]. For this reason, safety and security of assistive walking devices are of dramatic concern to senior citizen.

In addition, it is imperative to ensure that walking devices especially ICT ones which function based on human-made programs do not pose fall incidents to elderly on account of negligible errors in their programs. Besides, fall incidents give rise to another ethical issue which is responsibility of fall incidents. It is evident that assistive walking devices including fall detection ones play important role not only in the well-being of older adults but also in occurrence of fall incidents. For this reason, it is dramatic to identify the responsibility of such incidents.

Various types of tasks are made possible by making use of the services offered by autonomous service robots. Samples are taking care of old people at home [74] or accompanying guests in multi-level buildings [81].

Robotic service solutions include the simplest telepresence to the most complex functions to back caregivers. Examples are the Giraff (www.giraff.org) advanced in the ExCITE project [75], AVA (www.irobot.com/ava) and Luna [76], assisting needy persons in their everyday movements (www.aal-domeo.eu), self-management of long-lasting illness [77], comfort and safety as in the cases of Florence [78] and Robo M.D [79], and unification in an environment controlled by smart applications [80]. On the other hand, the number of robotic applications that are dedicated to social services in settings like smart office buildings is very few [81].

### Error and safety

Safety of elderly using assistive medical robots is of significant concern to older adults, their family members and caregivers, and robot designers and programmers. The assistive medical agent carries out a task in accordance with program(s) which is written by a range of codes through robot developers. For this reason, a negligible error in robot’s program might trigger older adults’ well-being and might cause fatal and severe consequences [82].

Technological care giving is already realized in most of Western European counties, but the technology that is usually used in this case is not robotic. On the opposite, some of it is no doubt low-tech. The aiding technology that is mostly available for old people in the UK ranges from portable alarms for requesting help; smoke, CO_2_ and flood sensors; pillboxes or containers that are designed in a way that let older people take their drug on time; fall sensors are another samples as well [83].

### Responsibility

In Ambient Intelligent Technology (AIT), artificial robot and its user interact with each other directly. This interaction amongst them has led to several issues such as responsibility of tasks, designation of control, decrease in human force, and allocation of decision-making [43, 45, 46, 82, 84]. In today’s world, artificial robots are increasingly and pervasively becoming autonomous which has resulted in diminishing human participation in some actions including decision-making. For this reason, liability of autonomous action is of critical concern [45, 85].

#### Responsibility concerns about robots for older adults

Robots are capable of interacting with human being and the encompassing environment in very intricate ways. The traditional theories of moral responsibility are challenged by social robots. The production of robots results in various ethical questions: what are the possible harmful consequences of such production? What would be the end of key moral concepts such as autonomy and privacy in a time when robots are integrated with human life? Are these robots moral agents? Is it ethical to take them responsible? These ethical issues result from the developing sovereignty of the smart technical products the most remarkable representatives of which are the social robots. Can robots be assumed as socially autonomous responsible confidant agents that care and, meanwhile, perform their duty as technical gadgets?

Whilst most of these concerns are related to other fields of engineering, the capacity of robots to turn into ethical agents puts forth another set of moral questions, such as those related to the rights and responsibilities of robots [86].

People’s ideas about the moral concerns introduced by autonomous products like robots very and address various notions such as the application of robots in, for instance, healthcare tasks. These views imply an understanding about the achievements of technology which depends on the ideas about the entity of technology and the relation of mind and matter in human and machine. The main focus of the usual approach of research in robot ethics deals with the robot and its entity and thoughts.

It helps to answer questions about the intelligence and rationality of robots, to see are they “moral agents”. Or, it restricts ethical concerns to things that, interactions with robots, might go wrong. For most of philosophers of morality, ethics is related to feeling of responsibility, the appropriateness of some one’s actions, and, then, the centrality of questions that consider moral status and action [87]. Usually, moral responsibility is only attributed to creatures that enjoy a tenable levee of moral agency—what does it mean—and concentrates on the suitability of what that agent performs, has performed, or can perform [88]. To investigate the ethics of robot technology, Coeckelbergh [88] puts forth an approach which centralizes human or interaction. Instead of thinking of a mental philosophy which regards the real entity and thought of robots, it would be better to adopt a philosophy of interaction and seriously consider the ethical importance of exterior form [89].

One of the benefits of the Accompany focus group’s discussions was the agreement that for monitoring the programming of robots, it is necessary to consider the communication of the older person who lived with a robot, with other organizations of formal and informal carers, instead of basically gratifying an aged person’s desires. Still, the data also propose that, at least, one approach—the “let’s do it together” strategy—may itself destabilize sovereignty by (unintentionally, perhaps) treating the older persons like children [83]. A robot would be considered as a social one when it takes responsibility, not when it is assigned with responsibility.

#### Human responsibility and robot responsibility

Robots have the power and ability of interacting with human being and human context in complicated ways. Robotics and making robots bring to the fore variety of applied ethical questions. Following introduces some of them: what are the potential risky consequences of making these robots? What autonomy and privacy concerns will be raised when robots turn to be an inseparable part of human life? Whilst most of these concerns are expressed in relation to other fields of engineering, the capability of robots to act as ethical entities introduces some other moral concerns, amongst them the right and responsibilities of robots?

The ethical issues have different layers that need to be discussed. The most central concerns deal with the responsibilities of robots [90–92] and human beings [92, 93].

There is a question shared by many people who are worried about this matter: who is responsible for the mistakes committed by robots? In cases that a robot does not pass the limits of autonomous function, a minimum level of the product liability is assumable. Given that robots follow the plan and procedure decided by some persons or companies, those people or companies are clearly responsible for failure (barring misuse). In the cases that robots are equipped with the accessories to be programmed by customers, the realm of liability will be clear. Still, in semi-autonomous robots such as self-driving cars, the concept of liability would be complicated, particularly when an accident happens in the cases of cooperation between robot and human agent.

In the cases that robot is autonomous, responsibility will be considered entirely as that of robot. It means that the robot is not under the direct influence of programs, programmers, or operators [94].

### Equal right for use of robot

It is found that one of the noticeable issues in robot ethics is having equal access to assistive medical robots. There have been a great number of debates surrounding this issue to consider whether it is affordable for every individual or particular group of individuals over the world to utilize and benefit from AIT or not [45, 95]. It is stated that unequal access to robots and healthcare systems might result injustice [47].

One of the ethical chief issues is having unequal access to assistive walking devices. In other words, it is injustice that particular groups of older adults because of different factors such as being from third-world countries do not benefit from assistive walking tools. In addition, it is pointed out that a noticeable number of senior citizens are strongly concern about the cost and also maintenance expenses of assistive walking device. Consequently, this factor slackens their interest towards use of walking devices [10, 58, 96, 97].

### Social impact

In some cases, use of assistive medical robots instead of weakening negative impacts, it strengthens the adverse effects such as social isolation which results in reducing social interaction [29, 82, 98]. The result of conducted research studies reveals that assistive robots such as telecare decline social communication [99]. In addition, Chan et al. [49] believed that smart home technology affects human’s relationship and communication with others owing to decreasing interaction between robot users and their caretakers.

It is found that albeit assistive walking devices such as wheeled walker compensate elderly’s disabilities in moving, yet there is a gap for amelioration to diminish fall incidents, whilst improving elderly’s appearance in public [8]. It is asserted that older adults encounter difficulties indoor and outdoor when they employ wheeled walkers. These issues take place when they move in curve, uphill, downhill, over obstacle(s), passing a door, on uneven ground, and carrying an object. In addition, mentioned issues might pose fall incidents to elderly. These issues might have negative effect on older adults’ morality and make them to feel embraced to carry out outdoor activities such as visiting medical doctors, using public transportation, and visiting family members or friend [8].

### Technology development

Over the past decades, there has been an abrupt development in technology. This has created hardship for technology users specifically older adults to learn and cope with new modern technology and systems. It is pointed out by Weiser and Brown [100] that it is significant for computer technology to be invisible when assisting users. In other words, technology users are not required to gain knowledge about technology. However, it is said that it is essential for technology users to be aware of advantages and disadvantages of technology’s role in their lives [101].

Apart from the mentioned ethical issues, there is another significant issue which the authors of this paper believe that it is essential to take this ethical issue into consideration and embed it in ethical framework of assistive medical robots. This issue is related to robots users’ feelings towards assistive robots. It is claimed that direct interaction between robots and individuals poses social isolation; therefore, this may influence robot users to have human feeling, namely love towards assistive robots. For this reason, it is important to consider appropriate standards in behaviours of robots to handle this issue.

Recently, there have been substantial technological developments in assistive walking devices. Some researchers believe that older adults are novice users; therefore, they prefer simple functions. Besides, older adults’ behaviour is towards emergency situation is different; they refuse to ask for assistance from their caretakers or nurses [102]. On the other hand, it is stated that some older adults found utilization of technology easy and convenient [103] [104]. It can be learned from literature review that there are common ethics issues between assistive walking devices and robots. Therefore, proper framework can be formed to alleviate and solve the ethical issues with the purpose of satisfying elderly’s needs.

## Discussion and conclusion

It is evident that assistive walking devices and robots play imperative role in senior citizens’ lives. These assistive agents and devices have embedded themselves into human’s daily tasks pervasively. It is obvious that robots increasingly have been empowered; therefore, the action of robots might have either destructive effect or useful impact on older adults. In other words, the consequence of assistive robot including walking device is far of significant concern rather than its action. In this case, the concept of consequentialism ethics theory can be applied in assistive walking devices and autonomous agent framework. Moreover, the common ethical issues of both assistive walking devices should be taken into consideration to complete a proper ethics framework which can be applied globally. In addition, a proper ethics framework play beneficial role to promote elderly’s standard of living, improve elderly’s satisfaction, compensate elderly’s disabilities, whilst reducing burdens of expenses related to healthcare services and centres.
